# Titration of analgosedation with neurally adjusted ventilatory assist in the ICU

**DOI:** 10.1186/cc9596

**Published:** 2011-03-11

**Authors:** MJ Sucre, A De Nicola

**Affiliations:** 1 San Leonardo Hospital, Castellammare di Stabia, Italy

## Introduction

The patient-ventilator asynchrony (PVA) is a cause of oversedation that prolongs mechanical ventilation unnecessarily. The current tools for measurement of sedation are inadequate for assessing the PVA. Neurally adjusted ventilatory assist (NAVA) is an innovative ventilatory mode that provides an excellent real-time monitor of the neural signal of diaphragmatic electrical activity (EAdi) and consequently highlights the PVA. Whether EAdi can be of help to titrate the level of sedation has not yet been proved, so we want to verify this conjecture. To titrate the level of analgosedation, we used this signal, which informs us continuously on changes in lung mechanics and synchrony.

## Methods

A prospective observational study on 50 coma patients, ventilated with Maquet SERVO-I, was performed, following monitoring chart EAdi and recording the numerical values of Edi peak and Edi min during the different ventilatory modes. We recorded the analgosedation via continuous infusion; the dose was titrated to achieve a score of the Richmond Agitation-Sedation Scale from -2 to +1 and the Behavioral Pain Scale ≤4.

## Results

The average duration of mechanical ventilation was 5.9 days (*P *= 0.004), the average of analgosedation was 4.8 days while the average length of stay was 6.4 days (*P *= 0.02). The average dose of remifentanil was varied between 0.075 ± 0.025 μg/kg/minute, propofol 0.5 ± 0.2 mg/kg/hour and clonidine 0.025 ± 0.02 μg/kg/minute. Comparing the pressure, volume and EAdi traces we identified all degrees of PVA. The Edi peak (16.8 ± 7.6 mV) and Edi min (0.1 ± 1.3 mV) values were used to adjust the level of sedation. The analgosedation quality was 97%.

## Conclusions

NAVA has been a real monitoring tool that provided a continuous dynamic lung overview. Monitoring NAVA avoided the more serious complications of the PVA: prolonged mechanical ventilation, barotrauma, and inadequate or excessive sedation. It was the only mode able to determine the asynchrony, allowing us to administer a tailored analgosedation, until the suspension. Moreover this protocol permitted us to save valuable resources. The measurement of PVA is a priority for the optimal sedation and NAVA can become an indicator for rating of analgosedation scales.
